# Tumor Heterogeneity of RCCs Assessed by mpMRI with Direct Radiological–Histopathological Correlation

**DOI:** 10.3390/diagnostics16132119

**Published:** 2026-07-07

**Authors:** Antonia M. Pausch, Viktoria S. Hadnagy, Toni Rabadi, Daniel Eberli, Niels J. Rupp, Andreas M. Hötker

**Affiliations:** 1 Diagnostic and Interventional Radiology, University Hospital Zurich, Raemistrasse 100, 8091 Zurich, Switzerland; 2 Department of Pathology and Molecular Pathology, University Hospital Zurich, 8091 Zurich, Switzerland; 3 Department of Urology, University Hospital Zurich, 8008 Zurich, Switzerland; 4 Faculty of Medicine, University of Zurich, 8091 Zurich, Switzerland

**Keywords:** RCC, mpMRI, pathology, biopsy, heterogeneity

## Abstract

**Background/Objectives:** The heterogenous nature of renal cell carcinomas (RCCs) is increasingly recognized. The purpose of this proof-of-concept pilot study was to evaluate correlations between multiparametric MRI (mpMRI)-derived and histopathological parameters in RCCs from spatially matched regions on both MRI and pathological examination to support targeted biopsy planning. **Methods:** In this prospective single-center pilot study, patients with solid renal tumors ≥2 cm undergoing nephrectomy were prospectively enrolled. Each patient underwent preoperative 3.0T-mpMRI including T2-weighted and pre-/post-contrast T1-weighted sequences, chemical-shift imaging, IVIM-DWI, and T1/T2*/R2 mapping. Tumor regions were defined jointly by a pathologist and radiologist, and identical regions of interest were assessed for each tumor region across all sequences to gain quantitative mpMRI-derived parameters. Histopathology provided quantitative regional fractions of viable tumor, fibrosis, hemorrhage, and cystic/necrotic components. Spearman’s rank correlations and univariable linear regression assessed associations between mpMRI and histopathological parameters on a regional level. **Results:** Across 49 tumor regions in eight patients (65.3% clear cell, 34.7% papillary RCCs), the mean viable tumor fraction was 80.9% (SD 17.6). The viable tumor fraction showed inverse correlations with nephrographic and delayed phase signal intensity changes (rho = −0.59/rho = −0.51), T1 values (rho = −0.56), true diffusion coefficient D (rho = −0.47), and ADC (rho = −0.45), and a positive correlation with R^2^ times (rho = 0.55). Delayed and nephrographic phase signal intensity changes (R^2^ = 0.41/R^2^ = 0.39) were the strongest single exploratory imaging correlates of viable tumor fraction. **Conclusions:** These findings support the feasibility of quantitative mpMRI parameters to capture regional intratumoral heterogeneity in RCCs, thereby highlighting regions with high viable tumor burden, which may help to refine the imaging-based assessment of RCCs in the future.

## 1. Introduction

The number of diagnosed renal tumors has been steadily increasing over the last decades, with more than 400,000 new cases of renal cell carcinoma (RCC) and about 155,000 deaths due to kidney cancer reported worldwide each year [1]. The 2022 WHO classification of renal tumors is based on a combination of morphological and molecular features. The most common subtypes are clear cell RCCs, papillary RCCs, and chromophobe RCCs [2]. While most patients present with a localized disease and may be treated curatively with partial or radical nephrectomy, approximately 30% present with either a locally advanced or metastatic disease and may require systemic therapy rather than surgery [3]. In both unresectable/metastasized disease, and in case of contraindications against surgical resection, biopsy of the tumor is indicated, except when watchful waiting is pursued as the management strategy. The tumor biopsy not only establishes histopathological confirmation of malignancy, but yields comprehensive information on tumor subtype and grade, thereby guiding further treatment decisions [4]. However, even within a single RCC entity, the significance of its wide morphological diversity is increasingly acknowledged [5]. For example, in clear cell RCCs (ccRCCs), subclonal genetic alterations, such as mutations in SETD2, PBRM1, and BAP1, as well as loss of chromosomes 14q and 9p, have been identified as significant contributors to tumor heterogeneity. These alterations are associated with variable prognosis and different responses to therapy [5]. Unfortunately, growing evidence indicates that, even when multiple cores are obtained, biopsy of a heterogeneous renal mass is susceptible to sampling error—especially in the assessment of tumor grade and genomic profile [6,7,8]. Therefore, identifying tumor regions with high viable tumor content would be highly valuable. A multiparametric MRI (mpMRI) is a non-invasive tool capable of providing detailed insights into tumor microenvironment by visualizing and quantifying various tissue parameters. In recent years, a variety of MRI techniques, e.g., to assess tumor microperfusion, diffusivity, or fat content, have been developed and applied to renal tumors, particularly for the differentiation of tumor subtypes and grades [9,10,11]. However, most previous analyses have relied on MRI parameters derived from either the entire tumor volume or a single, arbitrarily placed region of interest, without establishing a direct correlation between specific, histologically distinct tumor areas and their corresponding imaging features. Such information could inform the targeted selection of biopsy sites, particularly in patients with metastatic or locally advanced disease.

Given that the tumor entity is not known prior to biopsy, the primary aim of this proof-of-concept pilot study was to explore whether MRI can identify the RCC tumor regions on the mpMRI with the highest viable tumor content to inform MRI-guided biopsies. Accordingly, this study evaluated the feasibility of correlating the mpMRI-derived parameters with histopathological findings in RCCs from spatially matched regions on both imaging and pathological examination. As biopsy planning often proceeds without definitive tumor subtype knowledge, the inclusion of mixed RCC histologies was an intentional reflection of real-world clinical conditions.

## 2. Methods

### 2.1. Study Design

This prospective single-center pilot study (BASEC-ID: 2022-D0071), was approved by the institutional review board (Cantonal Ethics Committee Zurich). Consecutive patients aged 18 years or older with a solid renal tumor ≥2 cm, who were scheduled for surgical tumor resection and provided written informed consent, were enrolled. Exclusion criteria were contraindications against MRI (pacemaker, metallic implants, etc.), immunosuppressed patients (e.g., transplant patients), and a renal tumor as part of a suspected or confirmed genetic syndrome (e.g., Birt–Hogg–Dubé syndrome).

### 2.2. MRI Protocol

The MR scans were performed on 3.0 Tesla MR scanners (MAGNETOM Vida fit, Siemens Healthineers, Erlangen, Germany). Every patient underwent a standard clinical renal MRI and additional sequences for the purpose of this study, including T2-weighted sequences, pre- and post-contrast T1-weighted sequences with corticomedullary (C), nephrographic (N) and delayed phases, T1-weighted chemical shift sequences (in- and out-of-phase imaging), intravoxel incoherent motion (IVIM) diffusion-weighted sequences (b-values of 0, 20, 40, 170, 200, 250, 1500, and 2500 s/mm^2^), along with apparent diffusion coefficient (ADC) maps, and T1-, R2-, and T2*-mapping sequences. The T1, T2*, and R2 maps were automatically generated using the scanner-integrated processing pipeline from Siemens Healthineers (Erlangen, Germany), with direct access to the resulting parametric maps within the Picture Archiving and Communication System (PACS) workstation. IVIM parametric maps were computed using the MRI Body Diffusion Toolbox provided by Siemens Healthineers (Erlangen, Germany). This software performs advanced parametric modeling and enables voxel-wise estimation of diffusion-related parameters, including the true diffusion coefficient (IVIM_D in mm^2^/s) and the perfusion fraction (IVIM_fp).

### 2.3. MRI Analysis

Clinical renal MRIs were reviewed by board-certified radiologists as part of routine clinical care. For the purpose of this study, one radiologist with specialization in genitourinary radiology analyzed the mpMRI and study-specific sequences. Tumor regions on the MRI were defined in collaboration with a pathologist based on tumor tissue blocks. Regions of interest (ROIs, size ≥ 80 mm^2^) were then manually placed within these predefined tumor regions on corticomedullary phase images to encompass as much of the tumor as possible, using visual correlation in each case. Subsequently, using an automatic coregistration tool within the PACS (DeepUnity Diagnost, Dedalus, DH Healthcare GmbH, Bonn, Germany) or case-by-case visual adjustment, identical ROIs were mapped to the same tumor region on pre-contrast, nephrographic, and delayed phase images, on in- and opposed-phase sequences, on T1, T2*, and R2 maps, as well as IVIM parametric maps.

For the three post-contrast phases (corticomedullary (C), nephrographic (N), and delayed (D)), the signal intensity change *SI_ch_* was computed relative to the pre-contrast phase [12]: *SI_ch_*(%) = $\frac{SI_{post}-SI_{pre}}{SI_{pre}}\;\times \;100\;$ for *SI_post_* = *SI_ch_C_*, *SI_ch_N_*, or *SI_ch_D_*, accordingly. The chemical-shift index (*CS_index_*) was calculated using SIs of T1-weighted in- and opposed-phase images (*SI_in_* and *SI_opp_*) [9]: $CS_{index}\;\left(\%\right)=\frac{SI_{opp}-SI_{in}}{SI_{in}}\times \;100.$

### 2.4. Histopathological Workup

All surgical nephrectomy specimens (two partial and six radical nephrectomy specimens) were photographed both in their fresh state and after formalin fixation to document any macroscopic characteristics. The maximum tumor diameter was determined by correlating the macroscopic measurements with the preoperative MRI findings.

For the histological assessment, a full transverse slice through the plane of the largest tumor diameter was submitted for processing. This section included, when present, adjacent tumor-free renal parenchyma to allow for an evaluation of tumor boundaries and local tissue interactions. Hematoxylin- and eosin-stained slides were reviewed independently by two board-certified genitourinary pathologists.

Following diagnostic evaluation, all histological slides were digitally scanned for quantitative analysis. The following parameters were assessed based on tumor tissue blocks to allow for a separate evaluation of several tumor regions within each RCC:

• Tumor type/entity, classified according to current WHO criteria [2].
• Tumor grade, assigned according to WHO/ISUP grading [13].
• Quantitative morphometric analysis, performed through computer-assisted annotation of whole-slide images. The annotated features included total tumor area, extent of fibrosis, necrosis, hemorrhage, cystic components, and the presence of sarcomatoid or rhabdoid differentiation. The fraction of viable tumor as well as fibrotic, hemorrhagic, and cystic/necrotic components was expressed as a percentage of the whole tumor region.

**Figure 1:**
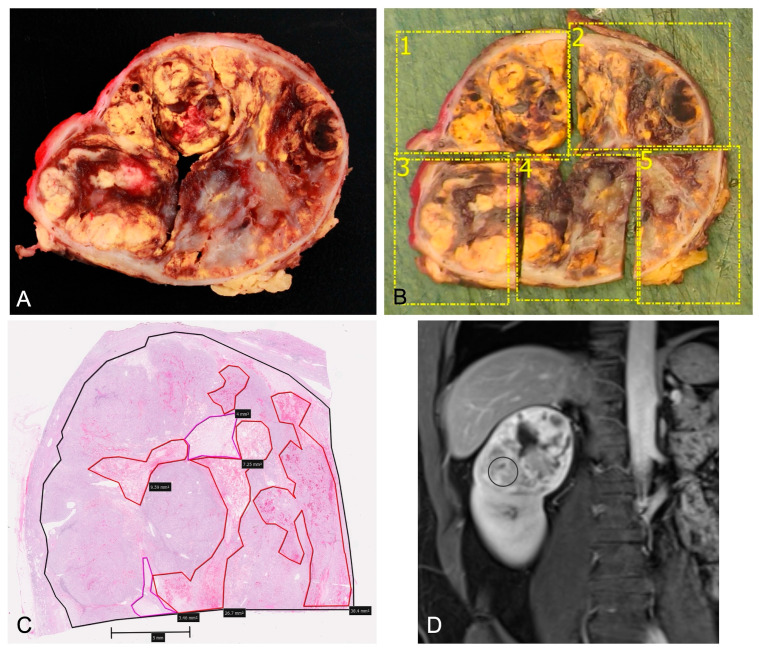
Surgical partial nephrectomy specimen of RCC in right kidney after formalin fixation (**A**) and subdivision into numbered tumor tissue blocks (**B**). Hematoxylin- and eosin-stained slide of tumor tissue block 3 with high viable tumor content (**C**); black outline: whole tumor area, violet: fibrotic components, red: hemorrhagic components. Corresponding representative ROI placement on the delayed phase of a post-contrast T1-weighted sequence in tumor tissue block 3 (**D**), illustrating how the MRI ROI was positioned to match the location of the corresponding histological tumor block.

Figure 1 depicts an example of the radiological–histopathological assessment.

### 2.5. Statistical Analysis

As the histologic tumor subtype is unknown prior to biopsy, the primary aim was to identify regions on the mpMRI with the highest viable tumor content, irrespective of RCC subtype. In this context, this study focused on a detailed, spatially matched analysis of multiple tumor regions per lesion. Because several regions were sampled within each tumor (49 regions from eight RCCs), observations were clustered within tumors and full hierarchical mixed-effects modeling was beyond the scope of this exploratory work. In particular, the small number of clusters (*n* = 8) would not allow for a stable estimation of the random-effects variance components. Consequently, *p*-values were interpreted descriptively, and emphasis was placed on effect sizes, namely Spearman’s correlation coefficients (rho), coefficients of determination (R^2^), and direction of regression slopes (β), to characterize the strength and direction of associations between the mpMRI-derived and histopathological parameters.

Spearman’s rank correlation was used to evaluate associations between the MRI parameters and the histopathological parameters, and univariable linear regression models were applied to estimate how much variance in the viable tumor content could be explained by each MRI parameter. To assess the consistency of associations on a regional level, regional mpMRI and histopathological measurements were also averaged on a per tumor level. Spearman’s rank correlations between the mean viable and fibrotic tumor fractions and the key mpMRI parameters (SI_ch_N_, SI_ch_D_, T1, R2, IVIM_D, ADC) were then recalculated on tumor-level mean values. All analyses were performed using the software R (version 4.5.1, [14]).

## 3. Results

### 3.1. Study Cohort

Nine patients were enrolled from August 2023 to November 2024. One patient had to be excluded due to significant motion artifact and consecutively low image quality. The final study cohort comprised a total of 49 tumor RCC regions in eight patients (median number of regions per tumor 5.5). The mean patient age was 60.5 years (SD 13.3), and the mean tumor diameter was 60 mm (SD 23 mm). Table 1 provides an overview of the characteristics of the tumor regions. No sarcomatoid or rhabdoid differentiation was identified.

### 3.2. Radiological–Histopathological Correlation

**Figure 2:**
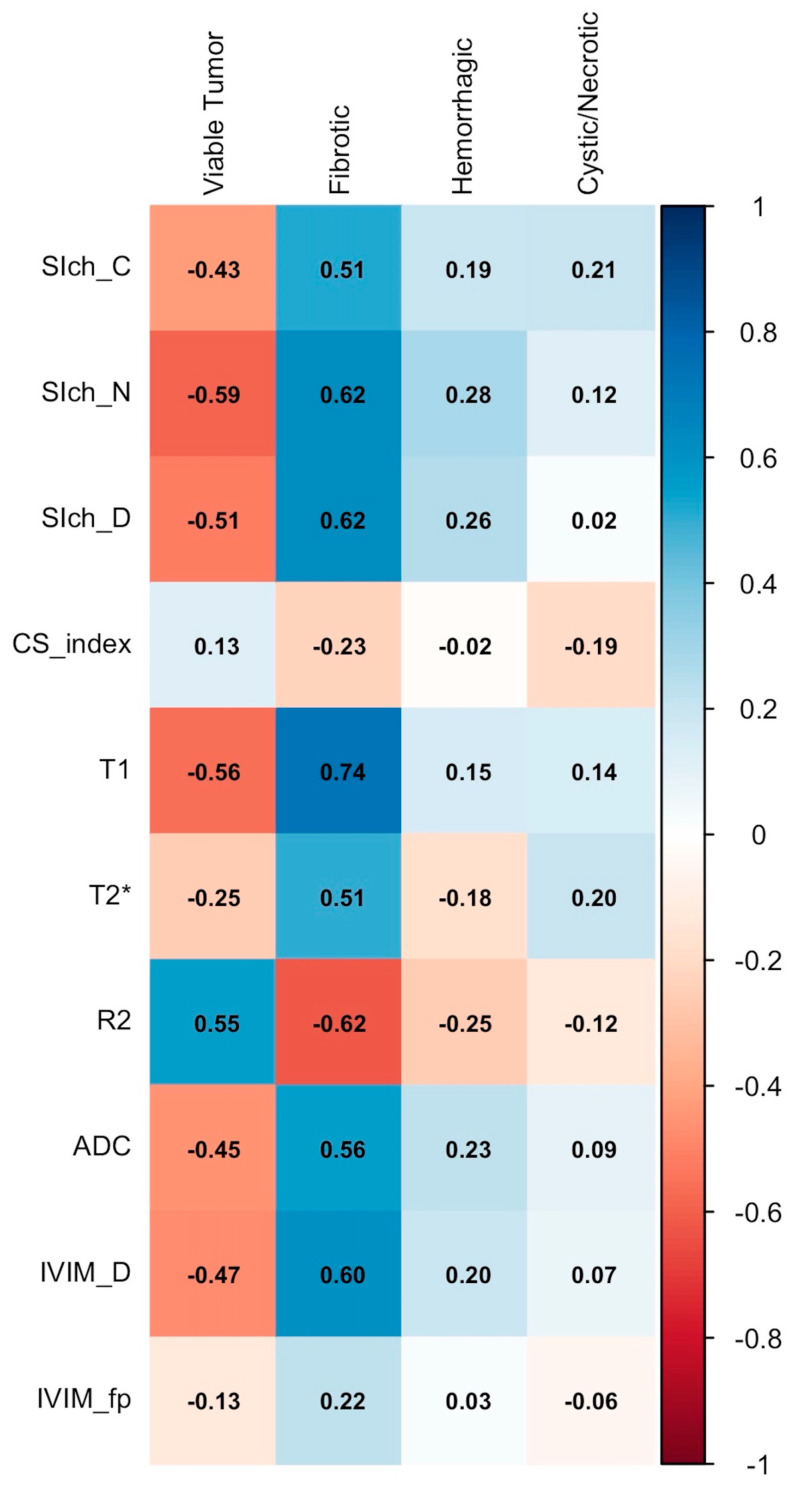
Heatmap of Spearman’s rank correlation coefficients (rho) between the mpMRI parameters and the histopathological component fractions. SI_ch_C_, SI_ch_N_, SI_ch_D_: signal intensity change (SI_ch_) for the three post-contrast phases (corticomedullary (C), nephrographic (N), and delayed (D)) relative to the pre-contrast phase. CS_index: chemical shift index. T1, T2*, R2: T1, T2*, R2 values. ADC: ADC map values. IVIM_D: true diffusion coefficient. IVIM_fp: perfusion fraction.

A heatmap of the Spearman’s rank correlation coefficients between the mpMRI parameters and the histopathological metrics is shown in Figure 2.

Overall, the viable tumor fraction showed the strongest inverse correlation to nephrographic-phase SI_ch_N_ (rho = −0.59), followed by T1 times (rho = −0.56) and delayed-phase SI_ch_D_ (rho = −0.51), while R2 times showed the strongest positive correlation (rho = 0.55). The IVIM parameters also correlated negatively with viable tumor content, with true diffusion coefficient (IVIM D; rho = −0.47), and apparent diffusion coefficient (ADC; rho = −0.45) values lower in regions of high viable tumors.

The fibrotic tumor component demonstrated a strong positive association with T1 times (rho = 0.74) and additional moderate positive correlations with SI_ch_N_ and SI_ch_D_ (rho = 0.62). No strong correlations were found for hemorrhagic and cystic/necrotic components.

The additional tumor-level-based analysis yielded correlation coefficients in the same direction and comparable magnitude, supporting the consistency of these associations (see Supplementary Table S1).

A univariable linear regression analysis (see Table 2) identified delayed-phase SI_ch_D_ (R^2^ = 0.41) as the strongest single exploratory imaging correlate of viable tumor fraction followed by nephrographic-phase SI_ch_N_ (R^2^ = 0.39) with a negative regression slope (−β) each.

Among the non-contrast parameters, R^2^ values demonstrated a positive relation with viable tumor fraction (R^2^ = 0.25, +β), therefore lower T2 times indicate greater viable tumor content. The T1 times, IVIM true diffusion coefficient, and ADC values each demonstrated inverse associations with viable tumor fraction (R^2^ = 0.22–0.25, −β), consistent with low T1 times and restricted diffusion in regions of high viable tumor.

## 4. Discussion

High-quality tumor biopsies of RCCs are crucial as they not only confirm malignancy but yield detailed histopathological insights and guide further treatment decisions [4].

This proof-of-concept pilot study of spatially matched mpMRI and histopathological specimens demonstrated that quantitative MRI parameters may capture intralesional heterogeneity of tumor viability on a regional level. Across all tumor regions, viable tumor fraction showed a consistent inverse correlation with relative signal intensity changes in nephrographic and delayed phases (SI_ch_N_: rho = −0.59, R^2^ = 0.39 and SI_ch_D_: rho = −0.51, R^2^ = 0.4). This indicates that regions with a lower delayed enhancement corresponded to a higher viable tumor content, which implies a positive association with washout and indicates that viable tumor preferentially exhibits rapid washout rather than persistent enhancement. Non-contrast parameters provided complementary information for biopsy planning. Consequently, higher R^2^ values (corresponding to shorter T2 times, rho = 0.55, R^2^ = 0.25) and lower T1 times (rho = −0.56, R^2^ = 0.25), as well as diffusion metrics (IVIM D: rho = −0.47, R^2^ = 0.23 and ADC: rho = −0.45, R^2^ = 0.22) were associated with a higher viable tumor content, consistent with increased cellularity in viable tumor tissue [15]. In addition, these non-contrast parameters might also represent a valuable non-invasive alternative for the assessment of tumor heterogeneity in patients with contraindications to gadolinium-based contrast agents. However, this remains a preliminary observation and warrants further validation in dedicated cohorts before clinical implementation. The T1 times (rho = 0.74) and relative signal intensity changes in nephrographic and delayed phases (SI_ch_N_ and SI_ch_D_: rho = 0.62), in turn, showed strong positive correlations with fibrotic components, as T1 hypointense fibrotic areas typically enhance slowly and retain contrast [16]. These findings underscore the ability of the mpMRI to differentiate viable tumor from fibrosis within the same lesion.

Tumoral heterogeneity is gaining increasing attention even within single entities, such as clear cell RCCs, which, despite its classic histological features of clear cytoplasm and a delicate capillary network, can exhibit substantial intratumoral variety [5]. Therefore, intratumoral heterogeneity is a well-recognized challenge in RCCs, as different tumor regions may harbor distinct histopathological features and may consequently limit the validity of single site biopsies [17]. Prior studies have mainly focused on global tumor assessment or radiomics signatures to distinguish RCC subtypes or grades [9,10,18,19,20], whereas this study demonstrates that several quantitative mpMRI parameters may also reflect regional variations in viable tumor content and other components, such as fibrosis, hemorrhage, or cystic/necrotic changes within an individual lesion. Our findings regarding enhancement patterns align with the study by Udayakumar et al. [21], who demonstrated that regional variations in MRI enhancement correlate with angiogenesis and inflammation pathways in clear cell RCCs. The application of IVIM imaging to assess intratumoral heterogeneity has been validated in several tumor types [15,22]. Yuan et al. [23] demonstrated that IVIM-derived parameters reflect heterogeneity of perfusion and diffusion within ccRCCs and correlate with tumor cellularity. Our observation of negative correlations between IVIM D, ADC, and viable tumor fraction aligns with these findings and supports the concept that restricted diffusion reflects increased cellularity in viable tumor regions.

From a clinical perspective, our data suggest that the mpMRI-derived parameters could potentially be used to guide targeted biopsies towards tumor regions with a high viable tumor burden, given that intratumoral heterogeneity can lead to undersampling of aggressive tumor components during percutaneous biopsy [17,24,25]. While the current data are not sufficient to define hard thresholds for clinical decision-making, they provide proof-of-concept that a quantitative mpMRI assessment carries biologically meaningful information on a regional level. Future, larger-scale studies should investigate how this approach can be extended to develop multivariable prediction models and to refine biopsy strategies, for example by applying radiomics-based techniques to identify the imaging predictors of most aggressive tumor areas.

Some limitations need to be acknowledged. This study was designed as a pilot with a limited number of tumors but a relatively large number of spatially matched regions. However, this constrained the ability to develop robust multivariable prediction models. The exploratory analysis should therefore be seen as hypothesis-generating rather than definitive, with an emphasis on effect sizes and direction of associations rather than formal predictive modeling. A further limitation is the lack of formal assessment of interobserver agreement for ROI placement, which may affect the generalizability of the ROI-based measurements. However, all tumor regions were defined in consensus by a radiologist and a genitourinary pathologist, which helped standardize ROI localization across cases and likely mitigated some variability. Tumor subtype represents another important confounding factor, as both clear cell and papillary RCCs were combined in the analysis. For instance, papillary RCCs are typically more hypovascular than clear cell RCCs and may exhibit distinct enhancement kinetics and baseline T1 values. Nevertheless, in clinical practice the tumor entity is not firmly established prior to biopsy, and the present study was designed to explore radiological–histopathological correlations irrespective of the subtype. In addition, partial-volume effects and sampling error on the histological side may have attenuated some associations.

## 5. Conclusions

This pilot study with direct radiological–histopathological correlation in RCCs supports the feasibility of using mpMRI-derived metrics to non-invasively assess tumor structure and to identify regions with high viable tumor burden. This approach may inform the design of future larger-scale trials to refine the imaging-based assessment of tumor biology prior to biopsy and treatment.

## Figures and Tables

**Table 1:** Tumor region characteristics.

Variable	Levels	Values
Tumor regions		49
Subtype (%)	ccRCC	32 (65.3)
	pRCC	17 (34.7)
WHO/ISUP grading (%)	1	2 (4.1)
	2	5 (10.2)
	3	42 (85.7)
	4	0 (0)
Mean percentage of tumor component	Viable tumor	80.9 (17.6)
(SD)	Hemorrhage	7.6 (12.5)
	Fibrosis	7.3 (12.8)
	Cystic/necrosis	4.3 (8.6)

SD: standard deviation; ccRCC: clear cell RCC; pRCC: papillary RCC.

**Table 2:** Univariable linear regression analysis to predict histopathological viable tumor fraction by the mpMRI parameters.

Parameter	R^2^	β
SI_ch_D_	0.41	−
SI_ch_N_	0.39	−
R2 map	0.25	+
T1 map	0.25	−
IVIM D	0.23	−
ADC	0.22	−

R^2^: coefficient of determination (proportion of variance explained). β: regression slope (positive values (+) indicate direct relationships, negative values (−) indicate inverse relationships).

## Data Availability

The data presented in this study are available upon request from the corresponding author upon approval of a methodologically sound proposal, receipt of appropriate ethical authorization, and completion of a data access agreement.

## Supplementary Materials

The following supporting information can be downloaded at: https://www.mdpi.com/article/10.3390/diagnostics16132119/s1, Supplementary Table S1: Spearman’s rank correlation coefficients (rho) between key mpMRI parameters and histopathological component fractions in the regional-level and tumor-level analyses.

## References

[1] Bray F., Laversanne M., Sung H., Ferlay J., Siegel R.L., Soerjomataram I., Jemal A. (2024). Global Cancer Statistics 2022: GLOBOCAN Estimates of Incidence and Mortality Worldwide for 36 Cancers in 185 Countries. CA Cancer J. Clin..

[2] Moch H., Amin M.B., Berney D.M., Compérat E.M., Gill A.J., Hartmann A., Menon S., Raspollini M.R., Rubin M.A., Srigley J.R. (2022). The 2022 World Health Organization Classification of Tumours of the Urinary System and Male Genital Organs—Part A: Renal, Penile, and Testicular Tumours. Eur. Urol..

[3] Tran J., Ornstein M.C. (2022). Clinical Review on the Management of Metastatic Renal Cell Carcinoma. JCO Oncol. Pract..

[4] Powles T., Albiges L., Bex A., Comperat E., Grünwald V., Kanesvaran R., Kitamura H., McKay R., Porta C., Procopio G. (2024). Renal Cell Carcinoma: ESMO Clinical Practice Guideline for Diagnosis, Treatment and Follow-Up. Ann. Oncol..

[5] Trpkov K., Hes O., Williamson S.R., Adeniran A.J., Agaimy A., Alaghehbandan R., Amin M.B., Argani P., Chen Y.-B., Cheng L. (2021). New Developments in Existing WHO Entities and Evolving Molecular Concepts: The Genitourinary Pathology Society (GUPS) Update on Renal Neoplasia. Mod. Pathol..

[6] Patel H.D., Johnson M.H., Pierorazio P.M., Sozio S.M., Sharma R., Iyoha E., Bass E.B., Allaf M.E. (2016). Diagnostic Accuracy and Risks of Biopsy in the Diagnosis of a Renal Mass Suspicious for Localized Renal Cell Carcinoma: Systematic Review of the Literature. J. Urol..

[7] Marconi L., Dabestani S., Lam T.B., Hofmann F., Stewart F., Norrie J., Bex A., Bensalah K., Canfield S.E., Hora M. (2016). Systematic Review and Meta-Analysis of Diagnostic Accuracy of Percutaneous Renal Tumour Biopsy. Eur. Urol..

[8] Garnier C., Ferrer L., Vargas J., Gallinato O., Jambon E., Le Bras Y., Bernhard J.-C., Colin T., Grenier N., Marcelin C. (2023). A CT-Based Clinical, Radiological and Radiomic Machine Learning Model for Predicting Malignancy of Solid Renal Tumors (UroCCR-75). Diagnostics.

[9] Hötker A.M., Mazaheri Y., Wibmer A., Karlo C.A., Zheng J., Moskowitz C.S., Tickoo S.K., Russo P., Hricak H., Akin O. (2017). Differentiation of Clear Cell Renal Cell Carcinoma From Other Renal Cortical Tumors by Use of a Quantitative Multiparametric MRI Approach. Am. J. Roentgenol..

[10] Henry R., Goetsch T., Brandhuber L., Labani A., Moliére S., Ohana M., Roy C. (2024). MRI Quantitative T1 and T2 Mapping of the Renal Cortex: Assessment of Normal Values and Potential Usefulness for Renal Masses at 3 T. Eur. J. Radiol..

[11] Woon D., Qin S., Al-Khanaty A., Perera M., Lawrentschuk N. (2024). Imaging in Renal Cell Carcinoma Detection. Diagnostics.

[12] Vargas H.A., Delaney H.G., Delappe E.M., Wang Y., Zheng J., Moskowitz C.S., Tan Y., Zhao B., Schwartz L.H., Hricak H. (2013). Multiphasic Contrast-Enhanced MRI: Single-Slice versus Volumetric Quantification of Tumor Enhancement for the Assessment of Renal Clear-Cell Carcinoma Fuhrman Grade. J. Magn. Reson. Imaging.

[13] Moch H. (2016). WHO-ISUP-Graduierungssystem für Nierenkarzinome. Pathologe.

[14] R Core Team (2025). R: A Language and Environment for Statistical Computing.

[15] Sigmund E.E., Cho G.Y., Kim S., Finn M., Moccaldi M., Jensen J.H., Sodickson D.K., Goldberg J.D., Formenti S., Moy L. (2011). Intravoxel Incoherent Motion (IVIM) Imaging of Tumor Microenvironment in Locally Advanced Breast Cancer. Magn. Reson. Med..

[16] Cheung H.M.C., Karanicolas P.J., Hsieh E., Coburn N., Maraj T., Kim J.K., Elhakim H., Haider M.A., Law C., Milot L. (2018). Late Gadolinium Enhancement of Colorectal Liver Metastases Post-Chemotherapy Is Associated with Tumour Fibrosis and Overall Survival Post-Hepatectomy. Eur. Radiol..

[17] Lim C.S., Schieda N., Silverman S.G. (2019). Update on Indications for Percutaneous Renal Mass Biopsy in the Era of Advanced CT and MRI. Am. J. Roentgenol..

[18] Ursprung S., Beer L., Bruining A., Woitek R., Stewart G.D., Gallagher F.A., Sala E. (2020). Radiomics of Computed Tomography and Magnetic Resonance Imaging in Renal Cell Carcinoma—A Systematic Review and Meta-Analysis. Eur. Radiol..

[19] Homayounieh F., Gopal N., Firouzabadi F.D., Sahbaee P., Yazdian P., Nikpanah M., Do M., Wang M., Gautam R., Ball M.W. (2024). A Prospective Study of the Diagnostic Performance of Photon-Counting CT Compared With MRI in the Characterization of Renal Masses. Investig. Radiol..

[20] Wang R., Zhong L., Zhu P., Pan X., Chen L., Zhou J., Ding Y. (2024). MRI-Based Radiomics Machine Learning Model to Differentiate Non-Clear Cell Renal Cell Carcinoma from Benign Renal Tumors. Eur. J. Radiol. Open.

[21] Udayakumar D., Zhang Z., Xi Y., Dwivedi D.K., Fulkerson M., Haldeman S., McKenzie T., Yousuf Q., Joyce A., Hajibeigi A. (2021). Deciphering Intratumoral Molecular Heterogeneity in Clear Cell Renal Cell Carcinoma with a Radiogenomics Platform. Clin. Cancer Res..

[22] Szubert-Franczak A.E., Naduk-Ostrowska M., Pasicz K., Podgórska J., Skrzyński W., Cieszanowski A. (2020). Intravoxel Incoherent Motion Magnetic Resonance Imaging: Basic Principles and Clinical Applications. Pol. J. Radiol..

[23] Yuan Q., Kapur P., Zhang Y., Xi Y., Carvo I., Signoretti S., Dimitrov I.E., Cadeddu J.A., Margulis V., Brugarolas J. (2016). Intratumor Heterogeneity of Perfusion and Diffusion in Clear Cell Renal Cell Carcinoma: Correlation with Tumor Cellularity. Clin. Genitourin. Cancer.

[24] Salami S.S., George A.K., Udager A.M. (2018). The Genomics of Renal Cell Carcinoma and Its Role in Renal Mass Biopsy. Curr. Opin. Urol..

[25] Nahouraii L.M., Allen J.L., Merrill S.B., Lehman E., Kaag M.G., Raman J.D. (2020). Histologic Heterogeneity of Extirpated Renal Cell Carcinoma Specimens: Implications for Renal Mass Biopsy. J. Kidney Cancer VHL.

