# Supplementary material for: Tumor Heterogeneity of RCCs Assessed by mpMRI with Direct Radiological–Histopathological Correlation
Source: Diagnostics (Basel). 2026 Jul 7;16(13):2119. doi: 10.3390/diagnostics16132119 (PMC13361544; doi:10.3390/diagnostics16132119)
Supplement: Supplementary file 1 [file diagnostics-16-02119-s001.zip › diagnostics-4329926-supplementary.pdf]

Histopathological component	mpMRI parameter	Regional level (rho)	Tumor level (rho)
**Viable tumor**	SIch_N	-0.59	-0.48
	SIch_D	-0.51	-0.40
	T1	-0.56	-0.36
	R2	0.55	0.36
	IVIM_D	-0.47	-0.38
	ADC	-0.45	-0.36
**Fibrosis**	T1	0.74	0.86
	SIch_N	0.62	0.71
	SIch_D	0.62	0.81

Supplementary Table S1. Spearman's rank correlation coefficients (rho) between key mpMRI parameters and histopathological component fractions in the regional-level and tumor-level analyses. SIch_N, SIch_D: signal intensity change (SIch) for the post-contrast phases (nephrographic (N) and delayed (D)) relative to the pre-contrast phase. T1, R2: T1, R2 values. ADC: ADC map values. IVIM_D: true diffusion coefficient.
